# Exosomal MALAT1 sponges miR-26a/26b to promote the invasion and metastasis of colorectal cancer via FUT4 enhanced fucosylation and PI3K/Akt pathway

**DOI:** 10.1186/s13046-020-01562-6

**Published:** 2020-03-24

**Authors:** Jingchao Xu, Yang Xiao, Bing Liu, Shimeng Pan, Qianqian Liu, Yujia Shan, Shuangda Li, Yu Qi, Yiran Huang, Li Jia

**Affiliations:** 1 College of Laboratory Medicine, Dalian Medical University, 9 Lushunnan Road Xiduan, Dalian, 116044 Liaoning Province China; 2 Department of General Surgery, The Second Affiliated Hospital of Dalian Medical University, Dalian, 116027 Liaoning Province China

**Keywords:** CRC, Exosomal MALAT1, FUT4, miR-26a/26b, PI3K/Akt/mTOR pathway

## Abstract

**Background:**

Exosomes are vesicles of endocytic origin released by various cell types and emerging as important mediators in tumor cells. Human metastases-associated lung adenocarcinoma transcript 1 (MALAT1) is a long non-coding RNA known to promote cell proliferation, metastasis, and invasion in colorectal cancer (CRC).

**Methods:**

The expression of MALAT1 was analyzed in CRC using qRT-PCR. FUT4 and fucosylation levels were detected in CRC clinical samples and CRC cell lines by immunofluorescent staining, western blot and lectin blot analysis. CRC derived exosomes were isolated and used to examine their tumor-promoting effects in vitro and in vivo.

**Results:**

The invasive and metastatic abilities of primary CRC cells were enhanced after exposure to exosomes derived from highly metastatic CRC cells, which increased the fucosyltransferase 4 (FUT4) levels and fucosylation not by directly transmitting FUT4 mRNA. Exosomal MALAT1 increased FUT4 expresssion via sponging miR-26a/26b. Furthermore, MALAT1/miR-26a/26b/FUT4 axis played an important role in exosome-mediated CRC progression. Exosomal MALAT1 also mediated FUT4-associated fucosylation and activated the PI3K/AKT/mTOR pathway.

**Conclusions:**

These data indicated that exosomal MALAT1 promoted the malignant behavior of CRC cells by sponging miR-26a/26b via regulating FUT4 and activating PI3K/Akt/mTOR pathway.

## Background

Colorectal cancer (CRC) is one of the leading causes of cancer-related morbidity and mortality [1, 2]. More than 60% of CRC patients have initiated the metastatic process by the time of diagnosis [3]. Although there are multiple tests available for CRC screening, each method has its own limitations in terms of sensitivity and specificity. To the best of our knowledge, carcinoembryonic antigen (CEA) and carbohydrate antigen 19–9 (CA19–9) are well established tumor markers with low sensitivity and specificity for early detection of CRC [4]. Hence, ideal CRC-specific biomarkers are urgently required to improve the current CRC diagnostic strategies.

Exosomes, membrane vesicles of endocytic origin ranging in size from 30 to 150 nm approximately, are emerging as key players in intercellular communication between cancer cells and their microenvironment [5]. A distinct feature of exosomes is that they efficiently carry and deliver molecular signatures (proteins, lipids, RNA and DNA) to recipient cells [6, 7]. In cancer development, exosomes are described as functional mediator of cancerous malignant alteration in recipient cells [8]. This intercellular communication is known to be involved in various pathophysiological processes including cell proliferation, migration, apoptosis, treatment resistance and metastasis [9–12], tumor innervation [13] and angiogenesis [14]. Exosomes also potentially participate in the development and progression of CRC. A recent study provides a novel notion that miR-200c and miR-141 contain in exosomes of mesenteric vein plasma could predict colon cancer patients with poor prognosis [15]. Exosomes derived from bone marrow-derived mesenchymal stem/stromal cells also enlarge the population of colon cancer stem cells by treating colon cancer cells (HCT-116, HT-29 and SW480) [16]. Therefore, it is important to explore the mechanisms by which exosomes derived from CRC cells regulate CRC progression, especially the metastatic process.

Long non-coding RNAs (lncRNAs) are RNA transcripts longer than 200 nucleotides that have limited or no protein-coding capacity [17]. Exosome-derived lncRNAs have been detected in a wide range of bodily fluids due to active cellular secretion [18]. Although ribonuclease is present in the blood, lncRNA nevertheless exists stably due to the protection of exosomes and microvesicles. Previous studies have demonstrated that exosome-derived lncRNAs affect tumor growth, metastasis, invasion, and prognosis by regulating the tumor microenvironment [19]. By traveling to cells through exosomes, lncRNAs could create a microenvironment suitable for the metastasis of tumor cells. Metastasis-associated lung adenocarcinoma transcript 1 (MALAT1) is an evolutionarily highly conserved lncRNA that lacks open reading frames. It plays essential roles in tumor development and is highly expressed in several tumors [20]. In CRC, inhibition of MALAT1 suppresses CRC progression and metastasis and improves the sensitivity of cancer cells to 5-FU [21]. Moreover, MALAT1 regulates the miR-106b-5p expression and further mediates the mobility of SLAIN2-related microtubules by functioning as a competing endogenous RNA, which results in the progression of CRC [22]. However, whether exosome-derived MALAT1 affects the malignant behavior of CRC cells by interacting with microRNAs (miRNAs) and mediating tumor metastasis is rarely reported.

In the present study, our findings provide further evidence that exosomal MALAT1 contributes to CRC progression and regulates FUT4 expression by sponging miR-26a/26b via fucosylation and PI3K/Akt pathway, which may provide novel insights into the function of exosomal MALAT1 in CRC.

## Materials and methods

### Clinical samples and cell culture

45 CRC tissues (24 with distant metastasis and 21 without metastasis) were collected from the First Affiliated Hospital of Dalian Medical University between 2015 and 2018. The study and its informed consent have been examined and certified by the Ethics Committee of the First Affiliated Hospital of Dalian Medical University (YJ-KY-FB-2016-16). Every patient was definitively identified as having CRC based on the clinicopathologic findings. None of the patients had received chemotherapy and/or radiotherapy prior to surgery.

Human CRC cell lines LoVo, HCT-8, SW620, and SW480 were obtained from KeygenBiotech Co. Ltd. (Nanjing, China). Cells were cultured in 90% DMEM/L-15 (Gibco, Grand Island, NY) supplemented with 10% heat-inactivated fetal bovine serum (Gibco) and 1% penicillin–streptomycin (HyClone, Logan, Utah, USA) at 37 °C with 5% CO_2_.

### Exosomes isolation from cell culture supernatants

Exosomes were collected from different CRC cells cultured in exosome-depleted fetal bovine serum (ultracentrifuged at 120,000 g overnight). Briefly, supernatant was collected from cells at approximately 80–85% confluence. After centrifugation of cells at 1000 g for 20 min, the supernatants were then centrifuged at 12000 g for 30 min to eliminate cells and debris. Finally, exosomes were obtained after centrifugation for 2 h at 120,000 *g* and then washed twice with a large volume of phosphate buffered saline (PBS). This protocol specifically collects exosomes and excludes large vesicles. The exosome proteins recovered were measured using the Bradford assay (Bio-Rad).

### Electron microscopy and exosome size and density measurement

Exosome suspension was placed onto 200 mesh carbon-coated grids and allowed to be absorbed to the velamen for 3 min. Then grids were allowed to dry at room temperature for 1 min and stained for contrast using 3% phosphotungstic acid. The samples were viewed with a JEM-2000EX transmission electron microscope (JEOL, Japan) and images were taken in a suitable proportion. The size and density of exosomes were measured by Zetasizer Nano (Malvern, England). Briefly, exosome-enriched pellets were resuspended in 1 ml of 0.1 μm triplefiltered sterile PBS. Three recordings of 60s were performed for each sample. Collected data were analyzed with Zetasizer Nano software, which provided size distribution report by intensity.

### Exosome labeling and macrophage trafficking in vitro

For exosome-tracking purposes, purified exosomes were fluorescently labeled using PKH67 (green) membranedye (Sigma-Aldrich, USA). Labeled exosomes were washed with PBS, re-collected by centrifugation at 12000 g for 30 min and then isolation with ExoQuick™. Labeled exosome pellets were resuspended in DMEM/L-15 medium and then added into receptor cell culture. After co-culturing for 3 h at 37 °C, the cells were washed with PBS for three times. Then the cells were fixed in 10% form aldehyde for 10 min and incubated with DAPI for 5 min. Images were obtained on a fluorescence microscope.

### RNA extraction and quantitative real-time PCR

Total RNA was isolated from frozen tissues and CRC cell lines, using the RNeasy Mini Kit (QIAGEN, Valencia, CA, USA), and cDNA was synthesized using QuantiTect Reverse Transcription Kit (QIAGEN, valencia, CA, USA) according to the manufacturer’s specifications. The expression of miRNAs was determined by using mirVanaTM qRT-PCR microRNA Detection Kit (Ambion Inc., Austin, TX, USA). Relative quantities of each miRNA were calculated using the ΔΔCt method after normalization with endogenous reference U6-small nuclear RNA. MALAT1 and FUT4 mRNA was quantified with SYBR-Green-quantitative real-time PCR Master Mix kit (Toyobo Co., Osaka, Japan). The expression level of MALAT1 and FUT4 was determined by using Biosystems 7300 Real-Time PCR system (ABI, Foster City, CA, USA) and calculated using the ΔΔCt method after normalization with GAPDH.

### Dual luciferase reporter gene assay

A pmirGLO Dual-Luciferase miRNATarget Expression Vector was purchased from GenePharma Co.Ltd. (Suzhou, China). Firefly luciferase functioned as primary reporter to regulate mRNA expression, and renilla luciferase was used as a normalized control. Co-transfection was conducted and the dual luciferase reporter assay system (Promega) was utilized. The mean luciferase intensity was normalized to renilla luciferase. Data were shown as the mean value ± SD and each experiment was performed thrice.

### RNA immunoprecipitation (RIP) assay

RIP assay was performed using the Magna RIP™ RNA Binding Protein Immunoprecipitation Kit (Millipore, Bedford, MA, USA). Cells were collected and lysed in complete RIPA buffer containing a protease inhibitor cocktail and RNase inhibitor. Next, the cell extracts were incubated with RIP buffer containing magnetic bead conjugated with human anti-Ago2 antibody (Millipore) or mouse immunoglobulin G (IgG) control. The protein was digested with proteinase K, and subsequently, the immunoprecipitated RNA was obtained. The purified RNA was finally subjected to a qRT-PCR analysis to demonstrate the presence of the binding targets.

### Western blot analysis and Lectin blot analysis

The tissues and cells were lysed in RIPA buffer with protease and phosphatase inhibitors (Roche, Beijing, China). Proteins (30 μg protein per lane) were resolved on 10% SDS-PAGE and transferred onto polyvinylidene difluoride membrane (Pall Corporation). Following protein transfer, membranes were blocked for 1 h in PBS containing 5% non-fat dry milk and 0.1% Tween-20. Blots were then incubated overnight with primary antibody or LTL lectin (1:500) (Vector Laboratories, Burlingham, CA) at 4 °C. Membrane proteins were detected by HRP-conjugated secondary antibody (1:1000) or HRP-labeled Streptavidin (1:1000) (Beyotime Biotechnology, Shanghai, China). GAPDH was used as a control. The proteins were visualized and quantified using a chemiluminescence method (ECL Pus Western Blotting Detection System; GE Healthcare UK Ltd., Buckinghamshire, UK) and the ImageQuant LAS 500 (General Electric Co, USA).

### Immunofluorescent staining

Paraffin-embedded sections (4 μm) were performed, and followed by antigen retrieval. The sections were washed in phosphate-buffered saline and incubated with primary antibodies including anti-FUT4 (1:150) at 4 °C overnight, and followed by an incubation with the secondary antibodies including Alexa Fluor 594-conjugated Goat Anti-Rabbit IgG (1:300) (Proteintech, Wuhan, China) and FITC-LTL lectin (1:500) (Vector Laboratories, Burlingham, CA) at 37 °C for 1 h. The sections were then counterstained with 4, 6 diamidino-2 phenyl-indole (DAPI; Sigma-Aldrich, St. Louis, MO, USA) for nuclear staining. Images were taken by a Carl Zeiss fluorescence microscopy (Carl Zeiss, Hallbergmoos, Germany).

Cell immunofluorescence staining was conducted after fixing cells with 10% formaldehyde for 40 min, and permeabilized with 0.3% Triton X-100. 2% BSA was utilized to block the non-specific binding for 30 min at room temperature. Then cells were incubated with the primary antibodies and secondary antibodies as mentioned above.

### Cell proliferation assay and focus formation assay

The Cell Counting Kit-8 (CCK-8; KeyGEN, Nanjing, China) and focus formation assay were conducted to determine the cell proliferation activity. Approximately 1 × 10^3^ cells per well were transferred to 96-well plates with 100 μl of DMEM medium containing 10% FBS and cultured in a humidified incubator at 37 °C for 24, 48, 72 and 96 h. The absorbance at 450 nm was measured following the addition of 10 μL of CCK-8 solution at 37 °C for 2 h. There were 5 replicates for each group, and 3 independent experiments were performed.

For the focus formation assay, cells (1 × 10^3^) were seeded in 6-well plates. The cultures were maintained in the DMEM containing 10% FBS, with medium changes every 3 days, until the appearance of foci from transformed cells was evident. Then the colonies were stained with 0.2% crystal violet, and foci were counted. Images of the colonies were obtained using a NIKON digital camera.

### Wound healing assay

Cells were cultured in serum-free medium and grown to 100% confluence in 6-well plates. After scratching the cell monolayer with a sterile pipette tip, the cells were washed twice with 1% PBS. The wound closing procedure was observed and photographed at 0 and 24 h under an inverted phase-contrast microscope (Olympus Corporation, Tokyo, Japan).

### Cell invasion assay

Cell invasion assay was performed using transwell inserts with polycarbonate membranes of 8.0-μm pore size (Corning Inc., NY) with ECMatrix gel (Chemicon) to form a continuous thin layer. In brief, 4 × 10^4^ cells in serum-free medium were added into the upper chamber. Culture medium with 10% FBS was added into the lower chamber. The chamber was cultured in 37 °C with 5% CO_2_ for 24 h and fixed with methanol. After staining with 0.4% crystal violet for 30 min, cells were photographed (400×) and counted in 5 random fields. Each experiment was performed thrice.

### Tumorigenicity assays in nude mice

4-week-old athymic male BALB/c nude mice were purchased from the Animal Facility of Model Animal Research Institute of Nanjing University (Nanjing, China). For xenograft models, the mice were randomly assigned to four groups. The mice in groups were inoculated subcutaneously with 1 × 10^7^ SW480 cells, SW480 + Exo-SW620 cells, SW480 + Exo-siRNA-SW620 cells and SW480 + Exo-siMALAT1-SW620 cells in the right flank. The size of tumor was measured every 7 days. 28 days after inoculation, mice were sacrificed and tumors were isolated and weighed. Tumor volume was calculated as the following formula: (length × width^2^)/2. Tumors were dissected out for tissue slice or proteins analysis.

### In vivo metastatic assays

Lung and hepatic metastatic model were used to measure cells metastatic ability, and the mice were randomly assigned to four groups as mentioned above. In brief, 2 × 10^6^ CRC cells in 0.2 ml PBS were injected into the tail vein of male BALB/c nude mice. At the beginning to show symptoms of dying after injection, tumor in lung metastasis was dissected out for tissue slice or proteins analysis. For liver metastatic model, nude mice were anaesthetized with pentobarbital sodium (Sigma, USA) by intraperitoneal injection. 1 cm incision was formed on the left side and the spleen was separated. Total of 2 × 10^6^ CRC cells were suspended in PBS and then injected into the spleen with a 30-gauge needle. After 5–6 weeks, the mice were sacrificed, and the spleen and liver were dissected out for tissue slice or proteins analysis.

### Statistical analysis

SPSS 17.0 software was used to analyze the experimental data. Each experiment was performed at least in triplicate and data were displayed as mean ± standard deviation (SD). Student’s t-test was used to compare the significant difference of two groups. The one-way analysis of variance (ANOVA) was used to determine the significant difference of multiple groups. *P* < 0.05 was considered to be statistically significant.

## Results

### Metastatic CRC cell-derived exosomes were transferred to primary CRC cells and increased malignant traits of primary CRC cells in vitro and in vivo

**Fig. 1:**
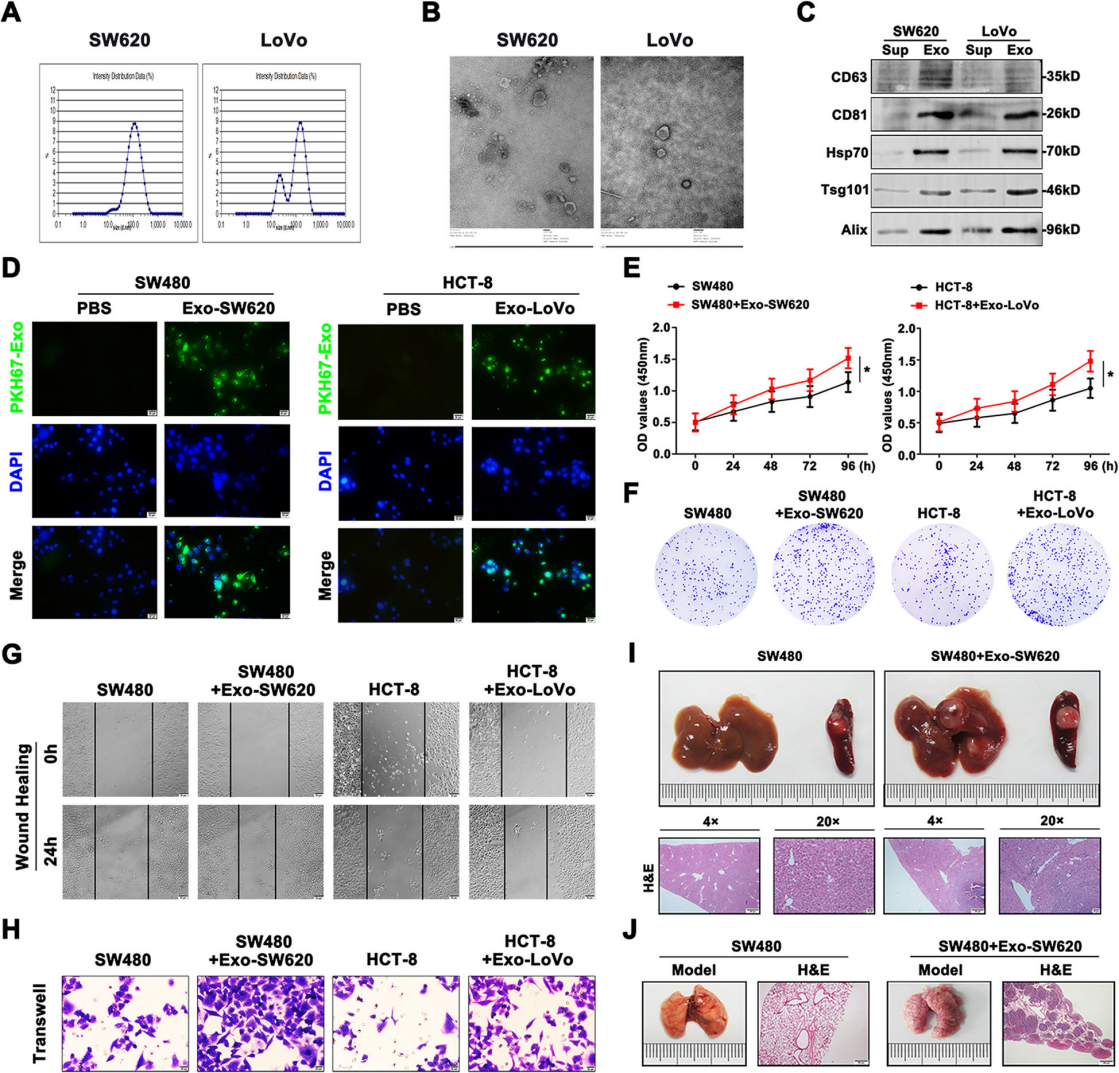
Metastatic CRC cell-derived exosomes were transferred to primary CRC cells and increased malignant traits of primary CRC cells. **a** The size of particles population ranged from 30 to 150 nm was confirmed by Zetasizer Nano analysis. **b** Electron micrograph images of purified exosomes from SW620 and LoVo cells were presented. **c** The symbolic markers of exsomes were detected by western blot from the supernatant and exosomes. **d** PKH67 staining was used to definite the internalization of exosomes in recipient cells. **e** CCK8 assays were carried out to determine the viability of treated CRC cell lines at 0, 24, 48, 72, and 96 h. **f** Colony formation assays were used to measure the proliferation of treated CRC cell lines. **g** Wound healing assay was carried out to show the migratory ability of treated CRC cells. **h** The invasion of differential treated CRC cells was verified by transwell assay. **i** Liver metastasis was conducted using SW480 cells with or without treatment of Exo-SW620. H&E staining was used to observe the morphological changes. **j** The lung metastatic model was established by SW480 cells treated or nontreated by Exo-SW620. H&E staining had been used as evaluation methods. Data were the means ± SD of triplicate determinants (*P < 0.05, **P < 0.01)

We first examined whether CRC cells secreted exosomes. Metastatic CRC cells SW620 and LoVo were cultured in exosome-depleted fetal bovine serum for 48 h and then we isolated exosomes using standard exosome isolation method. The Zetasizer Nano analysis confirmed a population of particles in the exosome size range of 30–150 nm (Fig. 1a). Exosome identity were confirmed by transmission electron microscope, Fig. 1b showed the images of exosomes and depicted rounded vesicles of ~ 100 nm, the expected exosome size range. The detection of characteristic CD63, CD81, HSP70, Tsg101 and Alix verified that the isolated particles were exosomes. CD63, CD81, HSP70, Tsg101 and Alix were also highly expressed in CRC cells-secreted exosomes than supernatant (Fig. 1c). To exclude potential interference of the subcellular fractions, the GM130 (Golgi marker), Calreticulin (endoplasmic reticulum marker) and Cytochrome c (mitochondria marker) levels were analyzed to confirme their absence in the isolated exosomes (Fig.S6).

The next step was to investigate whether metastatic CRC cells-secreted exosomes could be transferred and change malignant traits of primary CRC cells. We treated SW480 and HCT-8 cells with exosomes (10 μg per 1× 10^5^ cells) from cultured SW620 or LoVo cells for 48 h, and then examined cell function. The internalization of exosomes in recipient cells was visualized by using exosomes labeled with green fluorescent dye PKH67 (Fig. 1d). In vitro the experimental results showed that SW480 and HCT-8 cells were treated with exosomes, and proliferative rates, migratory and invasive capacity were all increased in two cell lines (Fig. 1e-h). Based on the promotional effects of exosomes on malignant phenotypes observed in vitro, we examined the potential of exosomes participated in CRC metastasis in vivo. Interestingly, we found that the nude mice bearing transfected SW480 cells treated with Exo-SW620 showed advanced liver metastasis, whereas no exosome treatment showed little liver metastasis (Fig. 1i). In addition, the same held true for the lung metastasis of SW480 cells treated with exosomes from SW620 cells, which facilitated the lung metastasis degree (Fig. 1j). Our findings highlight the presence of exosomal transfer between the CRC cells to spread malignant traits and the potential role of exosomes as main carriers of prometastatic factors.

### Exosomes increased FUT4 expression and fucosylation of CRC cells not by directly transmitting FUT4 mRNA

**Fig. 2:**
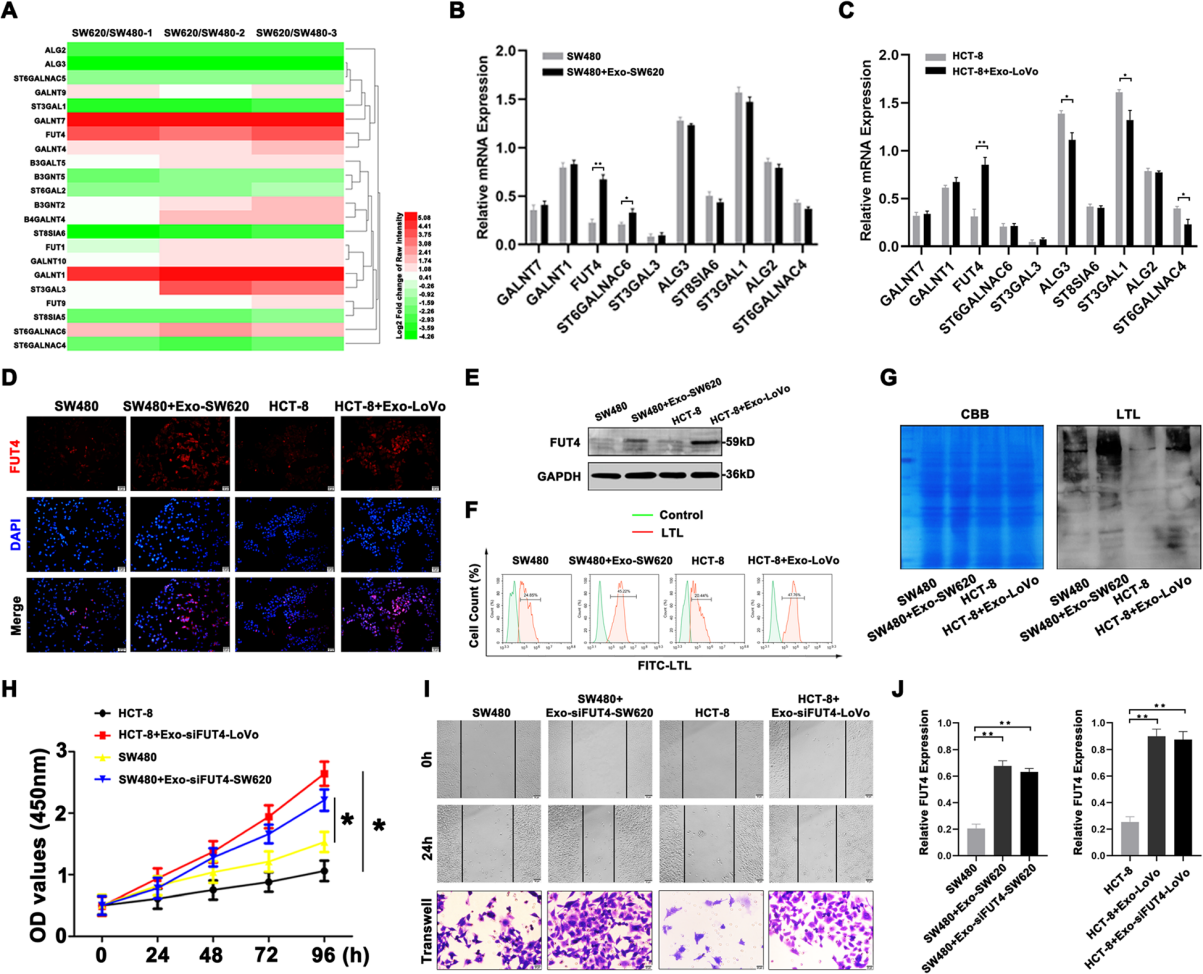
Exosomes increased FUT4 expression and fucosylation of CRC cells not by directly transmitting FUT4 mRNA (**a**) Glycosyltransferase genes in SW620 and SW480 cells were detected by Microarray analysis. **b** The expression of glycosyltransferase genes was shown in SW480 cells treated with Exo-SW620. **c** The expression of glycosyltransferase genes was shown in HCT-8 cells after treatment with Exo-LoVo. **d** Higher levels of FUT4 were determined in SW480 and HCT-8 cells treated with the exosomes from cultured SW620 or LoVo cells by immunofluorescence staining. **e** The level of FUT4 was verified by western blot. **f**, **g** The altered level of α1, 3-fucosylation was detected by FCM (**f**) and LTL blotting (**g**) analysis. **h** CCK8 assays were conducted to identify the viability of SW480 and HCT-8 cells treated with Exo-siFUT4-SW620 or Exo-siFUT4-LoVo. **i** Wound healing and transwell assays were used to determine the invasive and migratory ability of treated CRC cells. **j** FUT4 expression was analyzed in SW480 and HCT-8 cells treated with Exo-siFUT4-SW620 or Exo-siFUT4-LoVo. Data were means ± SD of three independent assays (*P < 0.05, **P < 0.01)

To explore the molecular mechanisms of exosomes-induced CRC metastatic process, the changes of gene expression after incubation with exosomes should be given special attention. Compared with SW480 cells, differentially expressed glycosyltransferase genes in SW620 cells were screened out (Fig. 2a). The expression of 5 genes (GALNT7, GALNT1, FUT4, ST6GALNAC6 and ST3GAL3) was up-regulated and 5 genes (ALG3, ST8SIA6, ST3GAL1, ALG2 and ST6GALNAC4) were down-regulated (Table S1). The expression of these glycosyltransferase genes was also determined in exosome treatment groups and untreated groups (Fig. 2b, c). Exosome treatment induced upregulation of FUT4 expression compared with exosome untreatment in SW480 cells or HCT-8 cells. In addition, the cells treated with exosomes from cultured SW620 or LoVo cells exhibited high protein levels of FUT4 (Fig. 2d, e). The α1, 3-fucosylation levels were also confirmed by LTL lectin blotting and FCM (Fig. 2f, g). These finding indicated that increased FUT4 expression and enhanced fucosylation might play potential roles in exosomes-related CRC metastasis.

We next investigated whether exosomes promoted FUT4 expression and fucosylation via directly transmitting FUT4 mRNA. SW620 and LoVo cells were transfected with siFUT4 and then used to isolate exosomes. Further investigation confirmed that exosomes from siFUT4-SW620 or siFUT4-LoVo cells could also promoted SW480 and HCT-8 cells proliferation, migration and invasion (Fig. 2h, i). Moreover, increased FUT4 expression was also detected in these two primary CRC cells after incubation with exosomes from siFUT4-mtastatic CRC cells (Fig. 2j). For further confirmation, we used another siRNA-2 to target FUT4 in SW620 and LoVo cells and then Exo-siFUT4–2-SW620 and Exo-siFUT4–2-LoVo were isolated to treat the primary CRC cells. The proliferative, invasive and migratory ability of SW480 and HCT-8 cells treated with Exo-siFUT4–2-SW620 or Exo-siFUT4–2-LoVo were also increased (Fig.S2A, B). In addition, the cells treated with exosomes from siFUT4–2-SW620 or siFUT4–2-LoVo cells also exhibited increased FUT4 expression, both in mRNA and protein levels (Fig.S2C, D). These demonstrated that exosomes could regulate FUT4 expression and fucosylation of recipient cells, but not by directly transmitting FUT4 mRNA.

### Exosomal MALAT1 sponged miR-26a/26b to increase FUT4 expression in primary CRC cells

**Fig. 3:**
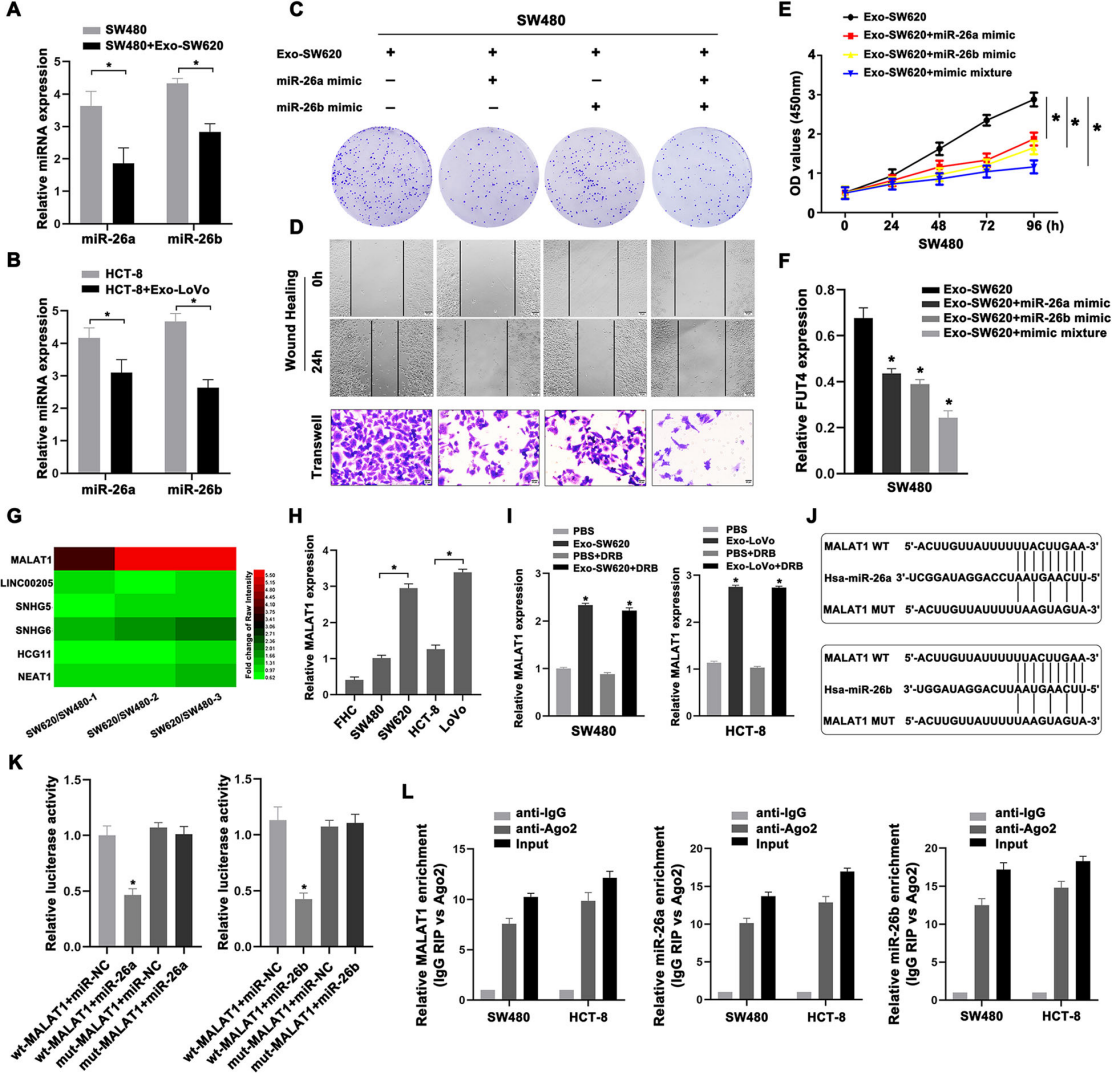
Exosomal MALAT1 sponged miR-26a/26b to increase FUT4 expression in primary CRC cells. **a** The expression of miR-26a and miR-26b was determined in SW480 cells treated with Exo-SW620. **b** The expression of miR-26a and miR-26b in HCT-8 cells was shown after treatment with Exo-LoVo. **c** The proliferative activity was measured by colony formation assay in SW480 cells treated with miR-26a/26b mimic in presence of Exo-SW620. **d** Wound healing and transwell assays were used to determine the migratory and invasive abilities of SW480 cells with different treatment. **e** CCK8 assay was carried out to measure the viability of treated SW480 cells. **f** FUT4 expression was determined in SW480 cells treated with miR-26a/26b mimic in presence of Exo-SW620. **g** Microarray analysis for six possible target lncRNAs of miR-26a and miR-26b in SW620 and SW480 cells. **h** Relative MALAT1 expression was determined by qRT-PCR in CRC cells. **i** MALAT1 levels were analyzed in SW480 and HCT-8 cells incubated with Exo-SW620 or Exo-LoVo for 24 h with or without DRB (15 mM). **j** Sequence alignment of miR-26a and miR-26b with the binding sites in the wild-type and mutant-type regions of MALAT1 was shown. **k** The relative luciferase activity of 293 T cells was tested after co-transfection with MALAT1 wide-type and miR-26a mimic or miR-26b mimic. **l** RIP assay was performed, and the co-precipitated RNA was subjected to qRT-PCR. RNA levels were presented as fold enrichment in Ago2 relative to IgG immunoprecipitates. Data were the means ± SD of triplicate determinants (*P < 0.05, **P < 0.01)

Exosome-induced FUT4 up-regulation was not by directly transmission of FUT4 mRNA, which made us considered the indirect involvement and mediation of other molecules in exosomes. In our previous study, increased FUT4 expression and enhanced aggressiveness of CRC cells could be regulated by inhibition of miR-26a/26b [23]. Hence we investigated whether miR-26a/26b was involved in exosome-mediated metastasis of CRC. As shown in Fig. 3a, b, miR-26a and miR-26b were all decreased in SW480 and HCT-8 cells treated with SW620 or LoVo exosomes, compared with that in control cells. We also enforced miR-26a/26b expression in SW480 cells pre-treated with Exo-SW620 by transfection with miR-26a/26b mimic. The results showed that exosome-enhanced cell proliferative rates, migratory and invasive capacities were attenuated by exogenous upregulation of miR-26a/26b (Fig. 3c-e). Meanwhile, exogenous overexpression of miR-26a and miR-26b decreased the mRNA levels of FUT4, which had been up-regulated by Exo-SW620 in SW480 cells (Fig. 3f). These data suggested that exosome-induced down-regulation of miR-26a/26b was essential to the progression of CRC.

To further explore the possible mechanisms of exosome-induced FUT4 expression by miR-26a/26b, we hypothesized exosomal lncRNAs might be emerged as essential regulators in these biological processes. CeRNA analysis and bioinformatics software (Starbase v3.0) were used to predict the potential lncRNAs binding sites in miR-26a and miR-26b. We found six possible target lncRNAs of miR-26a and miR-26b and identified their content change in CRC metastasis based on our previous Microarray analysis [24]. MALAT1 was the most obvious up-regulation among the six potential target molecules and might play a key role in regulating CRC development (Fig. 3g). Overexpression of MALAT1 was confirmed in metastatic CRC cell lines SW620 and LoVo, compared with primary CRC cell lines SW480 and HCT-8 (Fig. 3h). In addition, to confirm whether metastatic CRC cells-secreted MALAT1 could be transferred to primary CRC cells via exosomes, we quantified the MALAT1 levels in SW480 and HCT-8 cells treated with exosomes derived from cultured SW620 or LoVo cells. There was a significant increase in the cellular levels of MALAT1 in the recipient SW480 and HCT-8 cells following the exosomes treatment. We observed no effect of 5, 6-dichloro-1-(β-D-ribofuranosyl) benzimidazole (DRB), an RNA polymerase II inhibitor, on MALAT1 levels in the exosome-treated cells (Fig. 3i). We concluded that this increase in MALAT1 reflected exosome-mediated lncRNA transfer instead of MALAT1 endogenous expression in the recipient cells. In addition, it was also confirmed that MALAT1 resided in the lumen area of CRC exosomes. Exosomes pre-treated with RNase increased MALAT1 expression in the recipient SW480 and HCT-8 cells while exosomes with Triton pretreatment had no effect on MALAT1 transfer (Fig.S3A, B). According to the bioinformatic analysis, we determined the predicted binding sites. Dual-luciferase reporter gene assay confirmed that miR-26a and miR-26b were both the direct targets of MALAT1 (Fig. 3j, k). Argonaute 2 (Ago2) protein could bind miRNAs and lncRNAs. As shown in Fig. 3l, MALAT1, miR-26a and miR-26b all enriched in Ago2 pellet compared to the IgG immunoprecipitates. RIP assay illustrated that MALAT1 and miR-26a/26b existed in CRC cell lines, which corroborated the correlation between MALAT1 and miR-26a/26b. These results suggested that increased FUT4 expression was regulated by exosomal MALAT1 indirectly via sponging miR-26a/26b in CRC progression,.

### MALAT1 and exosomal MALAT1 were involved in clinical CRC progression

**Fig. 4:**
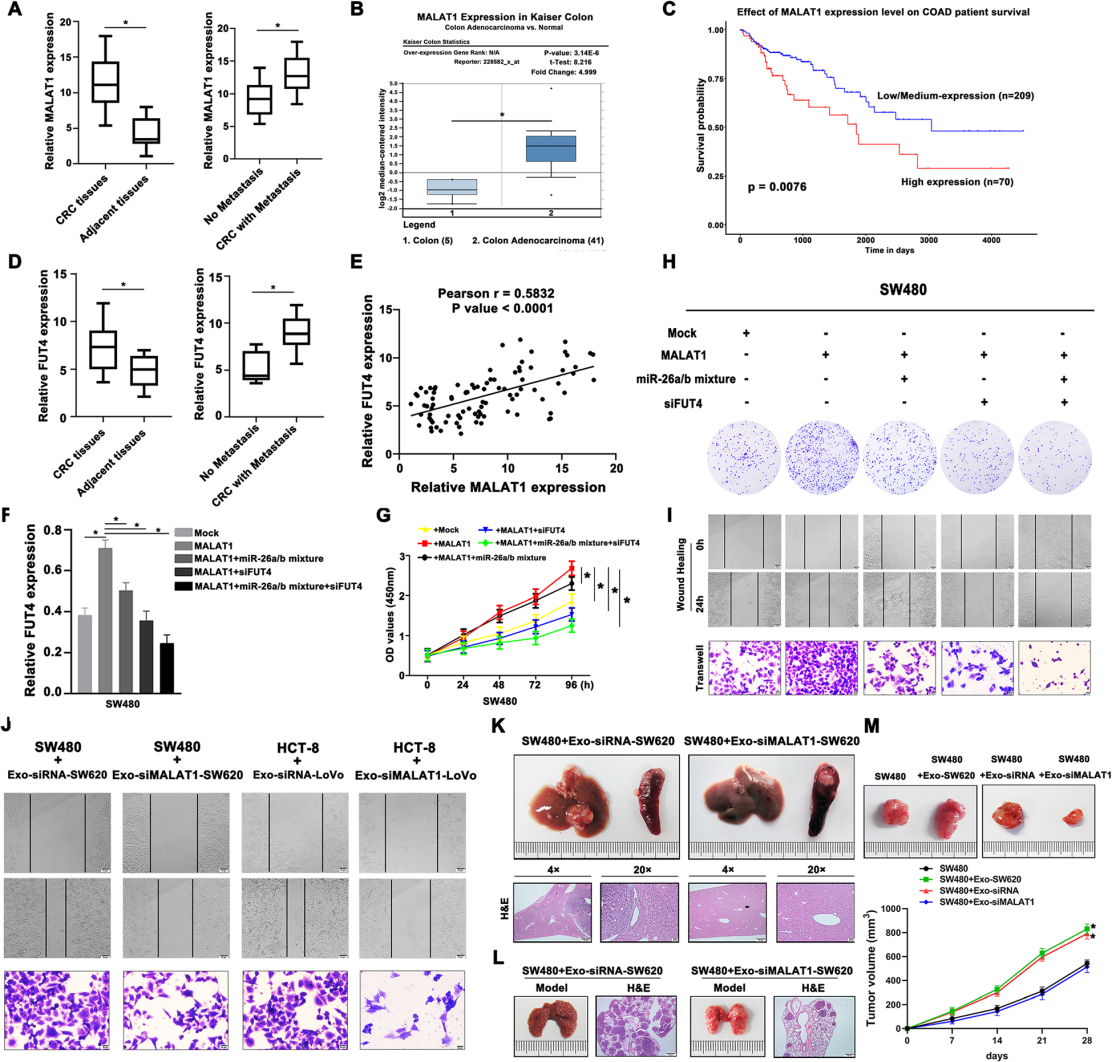
MALAT1 and exosomal MALAT1 were involved in clinical CRC progression (**a**) MALAT1 was overexpressed in CRC tissues than that in corresponding nontumor tissues and overexpression of MALAT1 was correlated with CRC metastasis. **b** The Oncomine boxed plot showed increased MALAT1 expression in colon adenocarcinoma (COAD) tissues using GEO database (GSE5206). **c** Kaplan–Meier survival analysis was illustrated on the basis of MALAT1 level using TCGA database. **d** FUT4 expression in CRC tissues was determined and the correlation between FUT4 expression and CRC metastasis was shown. **e** A positive correlation between MALAT1 and FUT4 was determined by Spearman’s correlation analysis. **f** Enhanced MALAT1 promoted FUT4 expression while treatment with miR-26a/26b mimic or FUT4 inhibitor reversed the stimulation. **g** CCK8 assay was carried out to measure the viability of treated SW480 cells. **h** The proliferative activity was measured by colony formation assay in SW480 with the treatment of MALAT1 mimic, miR-26a/26b mimic or FUT4 inhibitor. **i** Enhanced MALAT1 promoted the migratory ability while treatment with miR-26a/26b mimic or FUT4 inhibitor attenuated the enhancement effect. **j** Exosomes derived from siMALAT1-SW620 and siMALAT1-LoVo cells showed decreased effect on the migration and invasion in SW480 and HCT-8 cells compared with control. **k** Liver model was conducted in SW480 cells treated with Exo-siRNA-SW620 or Exo-siMALAT1-SW620. H&E staining was performed to evaluate the morphological changes. **l** Lung metastasis was conducted using SW480 cells treated with Exo-siRNA-SW620 or Exo-siMALAT1-SW620. H&E staining had been used as evaluation methods. **m** The xenograft model was established to show the carcinogenicity of SW480 and SW480 + Exo-SW620, SW480 + Exo-siRNA-SW620 and SW480 + Exo-siFUT8-SW620, the tumor growth curves were showed to trace the effect of exosomes on CRC progression. Data were means ± SD of three independent assays (*P < 0.05, **P < 0.01)

To understand the role of MALAT1 in CRC progression, we examined MALAT1 expression between 45 pairs of CRC tissues and the corresponding adjacent tissues. In accordance with the cell results, MALAT1 showed a higher level in tumor tissues and was also identified to connect with metastasis of CRC patients (Fig. 4a). To further evaluate the clinical significance of MALAT1 in cancer progression, we queried the Oncomine database (www.oncomine.com) to systematically assess the relative MALAT1 expression in different cancer types (cancer versus normal), which showed that MALAT1 was highly expressed in several tumors including CRC (Fig.S1A). The Oncomine boxed plot revealed significantly up-regulated MALAT1 in colon adenocarcinoma (COAD) tissues using GEO database (GSE5206) (Fig. 4b). Notably, as CRC is one of the most common type of human cancers, we also surveyed the RNA sequencing data of The Cancer Genome Atlas (TCGA) CRC study using UALCAN web-portal [25]. Overexpression of MALAT1 was found in COAD tumor samples and further clinical assess indicated that high MALAT1 expression was -significantly associated with advanced cancer stage (Fig.S1B, C). Kaplan–Meier survival analysis was then performed to compare the outcomes of patients dichotomized by MALAT1 expression. Patients with high MALAT1 expression level had a significantly worse survival probability than those with low MALAT1 expression (Fig. 4c). Then we detected the expression levels of FUT4 in clinical CRC samples in order to further clarify the relationship between MALAT1 and FUT4. Interestingly, higher FUT4 mRNA level was determined in CRC tumor tissues than the adjacent nontumor tissues and in cases of distant metastasis than those of no metastasis (Fig. 4d). Oncomine and TCGA database also revealed the increased FUT4 expression in CRC tumor tissues compared with normal tissues (Fig.S1D, E). Based on our results of clinical specimens, the positive correlation between MALAT1 and FUT4 was calculated (Fig. 4e), which was also proved by the data of Starbase v3.0 project (Fig.S1F).

To further investigate the effect of exosomal MALAT1-induced FUT4 up regulation and CRC development, the recipient SW480 cells were exogenously augmented by MALAT1 gene in the absence of exosome treatment. Overexpression of MALAT1 in SW480 cells could increase FUT4 expression and promote cell proliferation, plate colony formation, migration and invasion, but these enhanced effects were reversed by transfection with miR-26a/26b mimic or FUT4 inhibitor (Fig. 4f-i). In addition, silencing of MALAT1 was used to regulate the internalization of exosomal MALAT1. Exosomes lacking MALAT1 expression, derived from siMALAT1-SW620 and siMALAT1-LoVo cells, showed decreased effect on the migration and invasion in SW480 and HCT-8 cells (Fig. 4j). Moreover, the same effects were also verified in vivo experiments. SW480 cells pretreated with Exo-siMALAT1-SW620 showed both attenuated liver and lung metastasis, compared with Exo-siRNA-SW620 treatment groups (Fig. 4k, l). Tumorigenicity assays were also used to evaluate effects of exosome-induced tumor growth. While exosomes derived from metastatic cells promoted CRC tumor growth and the increased tumor masses were suppressed by targeting exosomal MALAT1 (Fig. 4m). In addition, exosomes intratumorally injection was shown to assess the promotional effect on CRC progression (Fig.S5). Mice injected with Exo-SW620 showed significant increase in tumor growth compared with mice injected with PBS, while intratumoral injection of Exo-siMALAT1-SW620 significantly attenuated the promotional effect on tumor growth. These results addressed exosomal MALAT1 was closely related to metastasis of CRC and targeting exosomal MALAT1 might be a treatment strategy for CRC development.

### Exosomal MALAT1 mediated FUT4-associated fucosylation and activated the PI3K/AKT/mTOR pathway in vitro

**Fig. 5:**
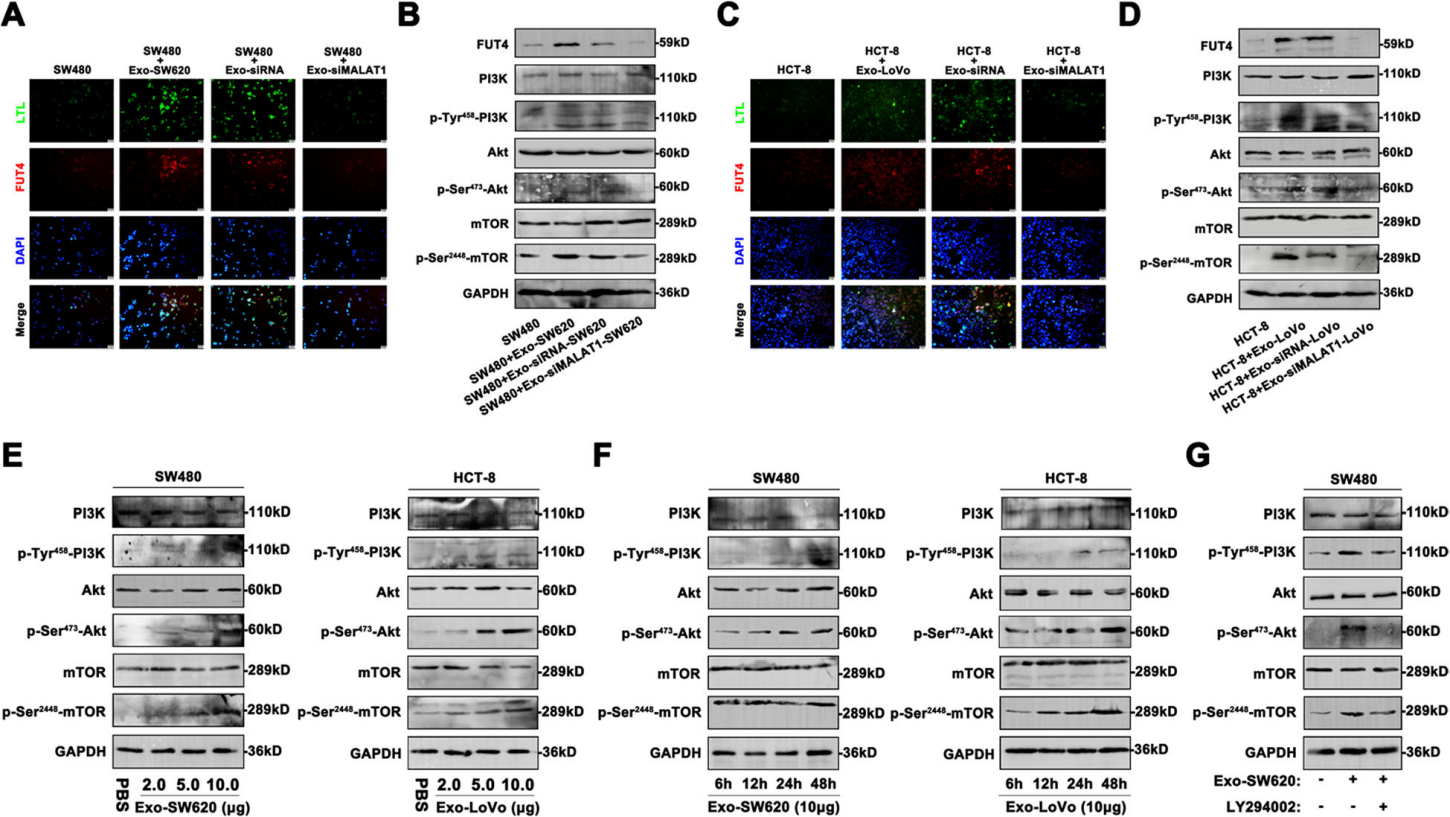
Exosomal MALAT1 mediated FUT4-associated fucosylation and activated the PI3K/AKT/mTOR pathway in vitro (**a**) Immunofluorescence staining was used to show the location of fucosylation and FUT4 in SW480 cells treated with Exo-SW620, Exo-siRNA-SW620 or Exo-siMALAT1-SW620. **b** The extracted proteins of SW480 cells with different exosomes treatment underwent western blot to assess the activity of PI3K/Akt/mTOR pathway. **c** HCT-8 cells treated with Exo-LoVo, Exo-siRNA-LoVo or Exo-siMALAT1-LoVo exhibited different levels of fucosylation and FUT4. **d** The activity of PI3K/Akt/mTOR pathway in HCT-8 cells with different exosomes treatment was measured by western blot. **e** SW480 and HCT-8 cells were planted in plates with or without different amounts of exosomes (2.0 μg, 5.0 μg, 10.0 μg) for 48 h and then the activity of PI3K/Akt/mTOR pathway was measured by western blot. **f** Western blot was used to measure the activity of PI3K/Akt/mTOR pathway in primary CRC cells treated with 10.0 μg exosomes at 6, 12, 24, and 48 h. **g** Exosomes treatment promoted the activity of the signaling in SW480 cells, while LY294002 reversed the stimulation

As a key enzyme of fucosylation, FUT4 performed significant alteration during CRC progression. Then we confirmed whether exosomal MALAT1 would promote fucosylation level of CRC cells by inducing FUT4 up regulation. Immunofluorescence staining was used to compare the expression level of α1, 3-fucosylation and FUT4 in CRC cell lines treated with exosomes. FITC labeled LTL, the α1, 3-fucosylated glycan-binding lectin, was used to identify fucosylation levels. The FUT4 and fucosylation levels were both increased in SW480 and HCT-8 cells treated with SW620 or LoVo exosomes, compared with that in control cells. However, these enhanced effects were attenuated by targeting exosomal MALAT1 (Fig. 5a, c). Our previous study revealed mTOR pathway was one of the most enriched pathways involved in the development of CRC. In order to figure out the molecular mechanism induced by exosomal MALAT1, the activity of PI3K/AKT/mTOR pathway was detected. The results showed that high levels of p-PI3K, p-Akt, and p-mTOR were observed in SW480 and HCT-8 cells treated with SW620 or LoVo exosomes, compared with exosome untreatment. However, SW480 cells treated with siMALAT1-SW620 exosomes showed weaker phosphorylation of PI3K/Akt/mTOR pathway and HCT-8 cells treated with siMALAT1-LoVo exosomes showed the same tendency (Fig. 5b, d). In addition, exosome-induced activation of PI3K/AKT/mTOR pathway exhibited dose- and time-dependent effects both in SW480 and HCT-8 cells (Fig. 5e, f). Treatment with PI3K inhibitor (LY294002) inhibited the phosphorylation of PI3K/Akt/mTOR of SW480 cells in present of Exo-SW620 (Fig. 5g). Moreover, FUT4 was closely related to the activity of PI3K/AKT/mTOR pathway. Upregulation of FUT4 could promote the activation of PI3K/AKT/mTOR pathway in SW480 cells with or without Exo-SW620 treatment, while targeting FUT4 attenuated these effects (Fig.S4A, B). All of the outcomes explained that exosomal MALAT1 could enhance FUT4-associated fucosylation and phosphorylation of PI3K/Akt/mTOR pathway, involved in the development of CRC.

### Promotional effects of exosomal MALAT1 on CRC metastasis and tumorigenesis via fucosylation and PI3K/AKT/mTOR pathway in vivo

**Fig. 6:**
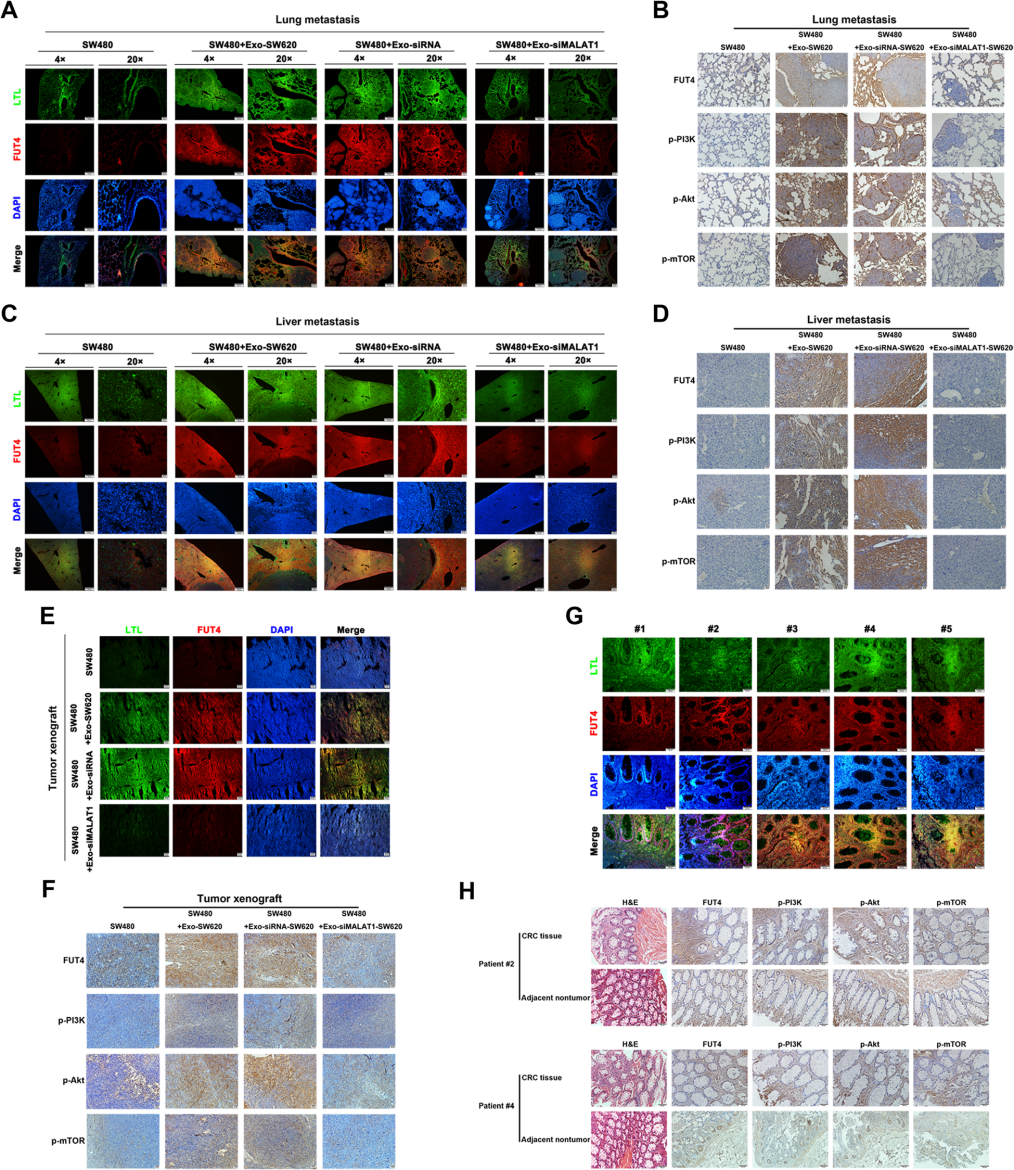
Promotional effects of exosomal MALAT1 on CRC metastasis and tumorigenesis via fucosylation and PI3K/AKT/mTOR pathway in vivo (**a**) The location and intensity of fucosylation and FUT4 were presented in the lung metastatic models established by SW480 cells with different exosomes treatment. **b** Immunohistochemistry staining was used to measure the activity of PI3K/Akt/mTOR pathway in lung metastatic models. **c** Liver metastasis was conducted using SW480 cells treated with different exosomes and LTL&FUT4 co-staining were completed to show the fucosylation levels. **d** The activity and influence of PI3K/Akt/mTOR pathway in liver metastatic models was measured by immunohistochemistry staining. **e** The levels of fucosylation and FUT4 in the xenograft models were detected by immunofluorescence staining. **f** The activation of PI3K/Akt/mTOR pathway was further determined in the xenograft models. **g** Immunofluorescence staining was used to measure fucosylation and FUT4 levels in CRC samples with metastasis. **h** Immunohistochemistry staining was used to measure the activity of PI3K/Akt/mTOR pathway in clinical sample 2 and 4, comparing the CRC tissue and adjacent nontumor tissue

As showed in Fig. 1 and Fig. 4, the nude mice bearing transfected SW480 cells treated with Exo-SW620 showed advanced liver and lung metastasis and increased tumorigenesis, while targeting exosomal MALAT1 attenuated the effect of exosome-mediated CRC metastasis and tumorigenesis. To further confirm whether fucosylation and PI3K/AKT/mTOR pathway were regulated in these processes, immunofluorescence staining and immunohistochemistry were used to validate the molecular mechanism in vivo. In accordance with the results in vitro, FUT4 associated fucosylation and the cellular signals including p-PI3K, p-Akt and p-mTOR in the exsomes-treated lung metastasis tissues were increased compared with those in the controls; However, MALAT1-knockdown exosomes failed to improve the fucosylation and phosphorylation levels of lung metastasis tissues compared to control **(**Fig. 6a, b). In addition, the same held true for the liver metastasis of SW480 cells treated with exosomes from SW620 cells, which facilitated fucosylation and activation of PI3K/AKT/mTOR pathway in liver metastasis tissues; On the contrary, MALAT1-knockdown exosomes attenuated these effects **(**Fig. 6c, d). Next, we found that subcutaneous inoculation of mixture of MALAT1-enriched exosomes with SW480 cells into nude mice promoted tumor growth and both the phosphorylation levels of cellular signals and fucosylation levels were increased in the exsomes-treated tumor tissues compared with those in the controls; However, MALAT1-knockdown exosomes failed to improve the fucosylation and phosphorylation levels of tumor tissue compared to control **(**Fig. 6e, f). Finally, we also examined the fucosylation and phosphorylation levels in clinical samples, results showed that the α1, 3-fucosylation levels were clearly detected in CRC samples with distant metastasis and the phosphorylation levels of cellular signals were higher in the CRC tissue compared to the adjacent nontumor tissue **(**Fig. 6g, h). These results addressed the efficient effect of exosomal MALAT1 on fucosylation and activation of cellular signaling for CRC progression in vivo.

## Discussion

Cancer-related deaths are primarily attributed to metastasis. Tumor microenvironment, a dynamic system mediated by intercellular communications, is responsible for tumor metastasis [26]. Therefore, it necessitates the research of the interaction between tumor and stroma modulated by exosomes. However, the functional role of fucosylation associated with exosomes in cancer progression is largely unknown. This study supported a new function of CRC derived exosomes in the transfer of MALAT1 to promote CRC cell aggressiveness by regulating FUT4-associated fucosylation and PI3K/Akt/mTOR pathway.

In recent years, many reports have convincingly demonstrated an important function of exosomes. Intercellular exchange of molecules via exosomes has been shown to be an effective mechanism of intercellular communication, especially within the tumor microenvironment [27]. Here, exosome-depleted FBS was used in our research mainly considering its advantages in cell nutritional support, maintaining good cellular secretion activity and the enrichment of exosomes. The exosomes treatment obviously promoted CRC cells proliferation, migration and invasion, as well as lung and liver metastasis, xenograft tumor growth. In line with our observations, Zeng et al. demonstrated that exosomes derived from CRC cells dramatically induced vascular leakiness and enhances CRC metastasis in liver and lung of mice [28].

Aberrant fucosylation and dysregulation of FUTs have been frequently found in human cancer [29, 30]. FUT4 could catalyze α1, 3-fucosylation that is particularly involved in a variety of pathological processes and in cancer biology [31]. Importantly, cancer-related CD15/FUT4 is overexpressed in most of metastatic colorectal cancer (mCRC) patients and participates in cetuximab or bevacizumab mechanisms of resistance in mCRC patients [32]. Similarly, we identified the profiles of FUT4 and α1, 3-fucosylation in mCRC patients and exosomes could regulate FUT4 expression and fucosylation level of recipient cells in CRC metastasis process, but not by directly transmitting FUT4 mRNA.

Growing evidence has indicated that the lncRNA MALAT1 contributes to tumor development in several types of human cancers, such as lung cancer and colorectal cancer [33, 34]. The elevated expression of MALAT1 plays important role in tumor cell progression. MALAT1 gene mutation was recently found in CRC and MALAT1 overexpression induced the invasion of SW480 cells [35]. Based on our results of clinical specimens and Oncomine and TCGA database, MALAT1 showed a higher level in tumor tissues and was connected with metastasis of CRC patients. In addition, competitive endogenous RNA (ceRNA) was reported that formed a large-scale regulatory network across the transcriptome. MALAT1 was overexpressed in six CRC cell lines and regulated the metastasis and invasion of CRC cells via targeting miR-20b-5p [21]. However, exosome-derived MALAT1 modulating CRC progression by interacting with miRNAs has not been addressed.

Exosomes potentially promote the malignancy of tumor cells by transferring oncogenic lncRNAs to induce tumor formation and metastasis [36]. Exosomal LncRNAs are transcribed to regulate the expression of oncogenes and tumor suppressor genes in tissue- and cell-specific manners [17]. Ren et al. quantitatively detected that carcinoma-associated fibroblasts promoted the stemness and chemoresistance of CRC by transferring exosomal lncRNA H19 [15]. Here, MALAT1 was proved to reside in the lumen area of CRC exosomes and exosomal MALAT1 derived from metastatic CRC cells could be transferred and regulate the expression of FUT4 in recipient cells, as a competing endogenous RNA for miR-26a and miR-26b, which regulated malignant traits of primary CRC cells. Furthermore, exosomes from siMALAT1-SW620 had no effect on promoting SW480 cells proliferation, migration and invasion, as well as lung and liver metastasis, xenograft tumor growth. On the other hand, the cell proliferation, migration and invasion were also attenuated in CRC cells treated with miR-26a/26b mimic or FUT4 inhibitor in presence of exosomal MALAT1. These results suggested that MALAT1/miR-26a/ 26b/FUT4 axis played an important role in exosome-mediated CRC progression and inhibition of this regulatory axis could attenuate the effect of exosome-induced CRC metastasis.

As a key oncogenic signaling pathway, PI3K/AKT/mTOR pathway plays a pivotal role in various cancers, including colorectal cancer [24, 37]. More researchers clarify the molecular mechanism of this signaling pathway involved in cancer proliferation, metastasis and chemoresistance [38, 39]. Based on our work, exosomes activated the PI3K/AKT/mTOR pathway in CRC. SW480 and HCT-8 cells treated with exosomes derived from SW620 or LoVo cells showed increased phosphorylation levels of PI3K/AKT/mTOR. However, targeting exosomal MALAT1 could attenuate this cellular signaling pathway. In addition, exosome-induced activation of PI3K/AKT/mTOR pathway exhibited dose- and time-dependent effects. Treatment with LY294002 inhibited the phosphorylation of PI3K/Akt/mTOR of SW480 cells in presence of Exo-SW620. Moreover, altered FUT4 regulated PI3K/AKT/mTOR pathway in exosome-induced CRC progression. These results further revealed that exosomal MALAT1 might promote CRC development through regulating FUT4 expression and PI3K/Akt/mTOR pathway, which offered a promising therapy target for CRC patients.

## Conclusion

In conclusion, we revealed the essential role of exosomes in communication between cancer cells and demonstrated that MALAT1 expression in exosomes was required for inducing aggressiveness phenotype by regulating FUT4-associated fucosylation and PI3K/Akt/mTOR pathway. Exosomal MALAT1 could be utilized as a diagnostic biomarker and therapeutic target for metastatic CRC.

## Supplementary information

**Additional file 1 Figure S1** MALAT1 and FUT4 expression in clinical CRC tissues based on the Oncomine and TCGA database (A) Malignant tumor diseases summary for MALAT1 expression (cancer versus normal) in Oncomine database. (B) Differential expression of MALAT1 was analyzed in colon adenocarcinoma based on TCGA database. (C) Expression of MALAT1 in COAD based on individual cancer stages. (D) Malignant tumor diseases summary for FUT4 expression (cancer versus normal) in Oncomine database. (E) TCGA samples were used for analyzing differential FUT4 expression between colon adenocarcinomas and adjacent normal tissues. (F) The positive correlation between MALAT1 and FUT4 was proved by the data of Starbase v3.0 project.

**Additional file 2 Figure S2** Exosomes lacking in FUT4 promoted CRC progression and increased FUT4 expression in recipient cells (A) Another siRNA was used to target FUT4 expression in CRC and CCK8 assays were conducted to identify the viability of treated CRC cells. (B) Wound healing and transwell assays were used to determine the invasive and migratory ability of SW480 and HCT-8 cells treated with Exo-siFUT4–2-SW620 or Exo-siFUT4–2-LoVo. (C) FUT4 mRNA level was analyzed in SW480 and HCT-8 cells treated with Exo-siFUT4-SW620 or Exo-siFUT4-LoVo. (D) FUT4 protein level was analyzed in SW480 and HCT-8 cells with treatment of different Exo-siFUT4-SW620 or Exo-siFUT4-LoVo. Data were means ± SD of three independent assays (*P < 0.05, **P < 0.01).

**Additional file 3 Figure S3** MALAT1 resided in the lumen area of CRC exosomes (A) MALAT1 levels were analyzed in SW480 cells incubated with Exo-SW620 pretreated with RNase or Triton. (B) MALAT1 levels were analyzed in HCT-8 cells incubated with Exo-LoVo pretreated with RNase or Triton. Data were means ± SD of three independent assays (*P < 0.05, **P < 0.01).

**Additional file 4 Figure S4** Altered FUT4 mediated PI3K/AKT/mTOR pathway in CRC cells (A) FUT4 or siFUT4 regulated the activity of PI3K/Akt/mTOR pathway by western blot. (B) The activity of PI3K/Akt/mTOR pathway was measured in SW480 cells treatment with Exo-SW620, FUT4 or siFUT4.

**Additional file 5 Figure S5** The effect of intratumorally injected exosomes on CRC tumor growth (A) The mice models were randomly assigned to four groups and inoculated subcutaneously with equal number of SW480 cells. Then from the seventh day on, PBS or 10 μg exosomes were intratumorally injected into tumor-bearing mice twice a week. 21 days after injection, mice were sacrificed and tumors were isolated and weighed. (B) The tumor size was measured every 7 days and tumor volume was calculated to assess the promotional effects of exosomes on CRC progression. Data were means ± SD of three independent assays (*P < 0.05). 9.

**Additional file 6 Figure S6** The protein levels of GM130, Calreticulin and Cytochrome c in exosomes from CRC cells and cell lysates were analyzed. Western blot was used to detect GM130 (Golgi marker), Calreticulin (endoplasmic reticulum marker) and Cytochrome c (mitochondria marker) expression in CRC exosomes and cell lysates with equivalent protein amount.

**Additional file 7.**

**Additional file 8.**

## Data Availability

The datasets used and/or analyzed during the current study are available from the corresponding author on reasonable request.

## Abbreviations

CRC: Colorectal cancer
FUT4: Fucosyltransferase 4
MALAT1: Human metastasis-associated lung adenocarcinoma transcript 1
PI3K: Phosphatidylinositol 3-kinase
CEA: Carcinoembryonic antigen
CA19–9: Carbohydrate antigen 19–9
miRNAs: microRNAs
COAD: Colon adenocarcinoma
FUTs: Fucosyltransferases
GlcNAc: N-acetylglucosamine
lncRNAs: Long non-coding RNAs
RIP: RNA immunoprecipitation
FCM: Flow Cytometry
EM: Electron microscopy
TCGA: The Cancer Genome Atlas
ceRNA: competitive endogenous RNA
