# Supplementary figures and images for: Exosomal MALAT1 sponges miR-26a/26b to promote the invasion and metastasis of colorectal cancer via FUT4 enhanced fucosylation and PI3K/Akt pathway
Source: J Exp Clin Cancer Res. 2020 Mar 24;39:54. doi: 10.1186/s13046-020-01562-6 (PMC7092616; doi:10.1186/s13046-020-01562-6)

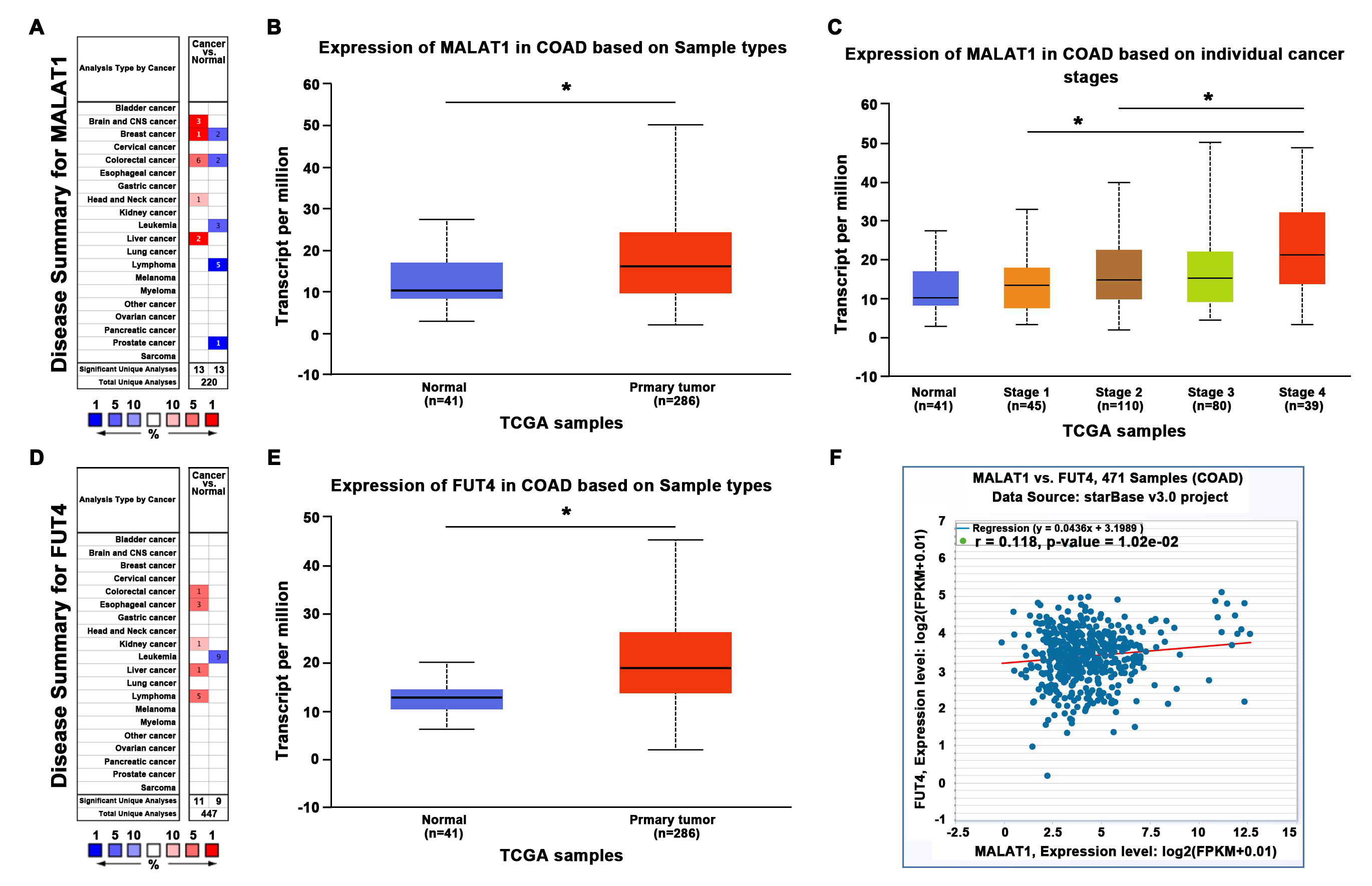

Supplement: Supplementary file 1 — Additional file 1 Figure S1 MALAT1 and FUT4 expression in clinical CRC tissues based on the Oncomine and TCGA database (A) Malignant tumor diseases summary for MALAT1 expression (cancer versus normal) in Oncomine database. (B) Differential expression of MALAT1 was analyzed in colon adenocarcinoma based on TCGA database. (C) Expression of MALAT1 in COAD based on individual cancer stages. (D) Malignant tumor diseases summary for FUT4 expression (cancer versus normal) in Oncomine database. (E) TCGA samples were used for analyzing differential FUT4 expression between colon adenocarcinomas and adjacent normal tissues. (F) The positive correlation between MALAT1 and FUT4 was proved by the data of Starbase v3.0 project. [file 13046_2020_1562_MOESM1_ESM.tif]

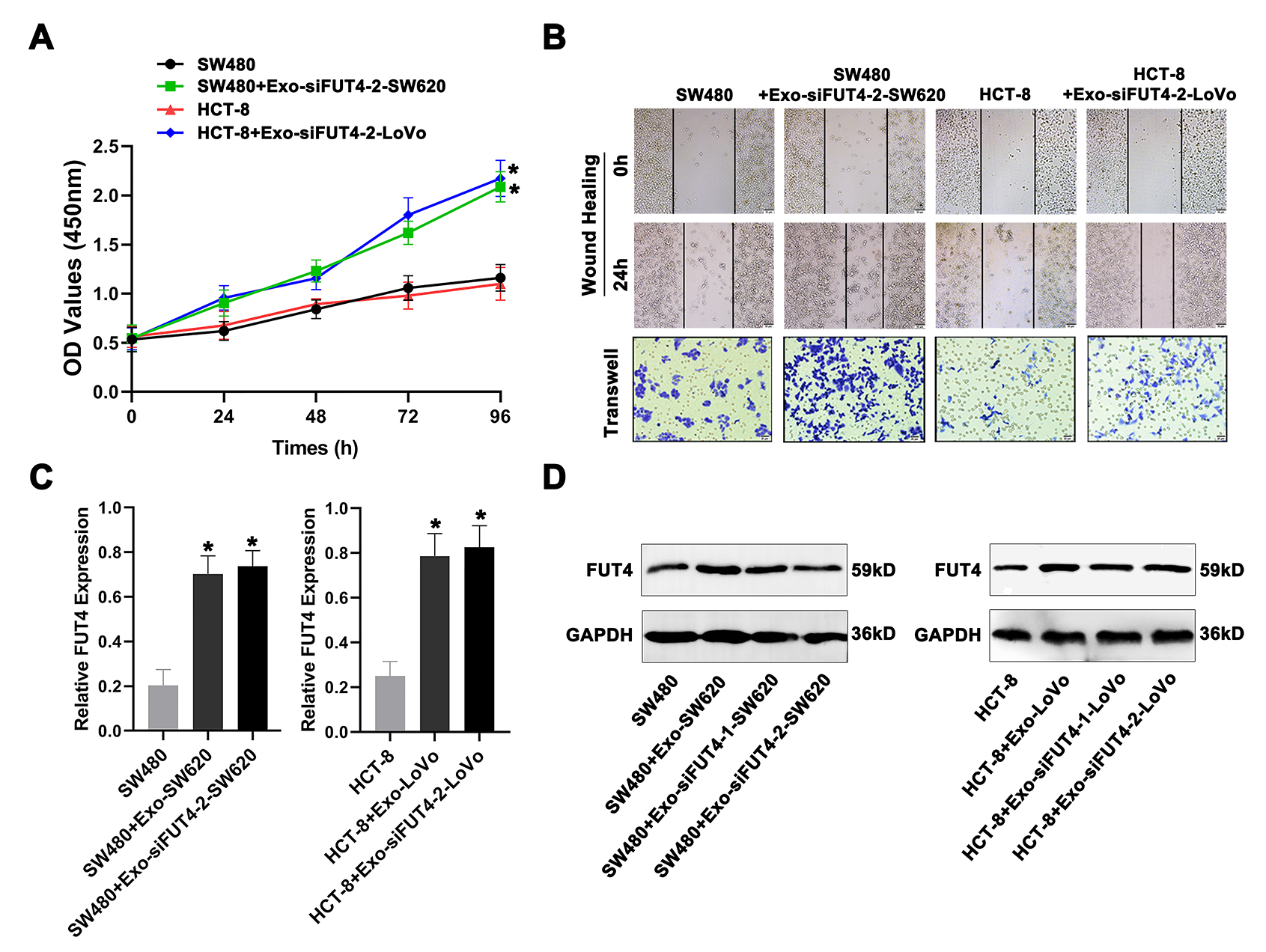

Supplement: Supplementary file 2 — Additional file 2 Figure S2 Exosomes lacking in FUT4 promoted CRC progression and increased FUT4 expression in recipient cells (A) Another siRNA was used to target FUT4 expression in CRC and CCK8 assays were conducted to identify the viability of treated CRC cells. (B) Wound healing and transwell assays were used to determine the invasive and migratory ability of SW480 and HCT-8 cells treated with Exo-siFUT4–2-SW620 or Exo-siFUT4–2-LoVo. (C) FUT4 mRNA level was analyzed in SW480 and HCT-8 cells treated with Exo-siFUT4-SW620 or Exo-siFUT4-LoVo. (D) FUT4 protein level was analyzed in SW480 and HCT-8 cells with treatment of different Exo-siFUT4-SW620 or Exo-siFUT4-LoVo. Data were means ± SD of three independent assays (*P < 0.05, **P < 0.01). [file 13046_2020_1562_MOESM2_ESM.tif]

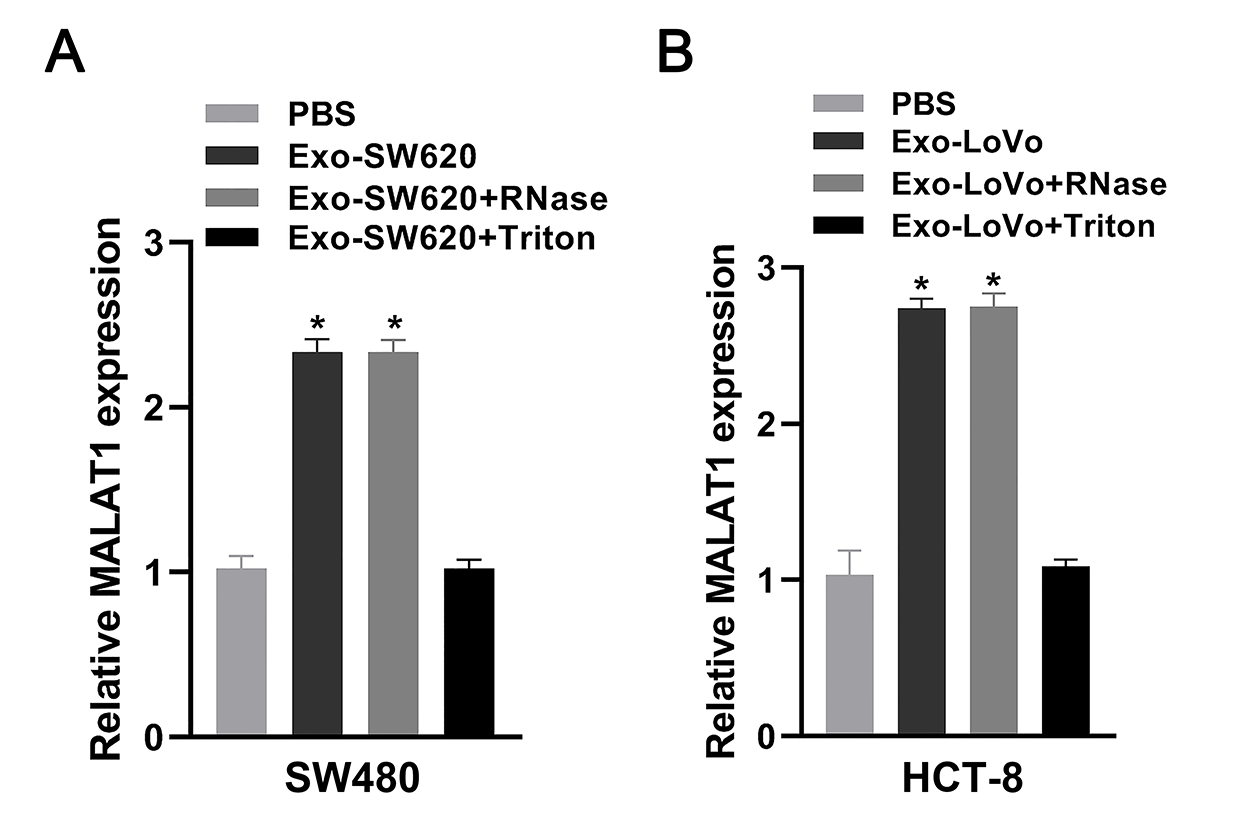

Supplement: Supplementary file 3 — Additional file 3 Figure S3 MALAT1 resided in the lumen area of CRC exosomes (A) MALAT1 levels were analyzed in SW480 cells incubated with Exo-SW620 pretreated with RNase or Triton. (B) MALAT1 levels were analyzed in HCT-8 cells incubated with Exo-LoVo pretreated with RNase or Triton. Data were means ± SD of three independent assays (*P < 0.05, **P < 0.01). [file 13046_2020_1562_MOESM3_ESM.tif]

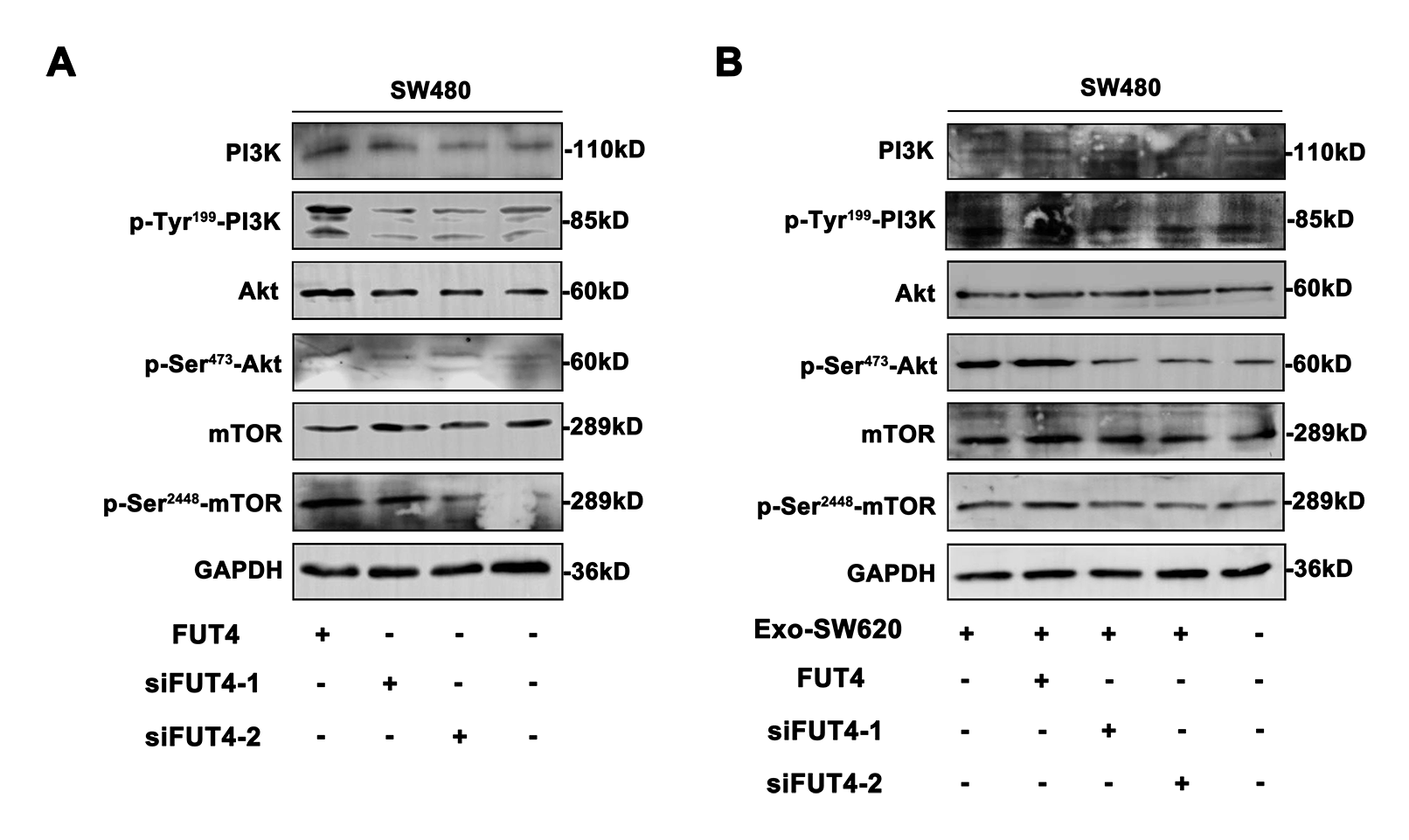

Supplement: Supplementary file 4 — Additional file 4 Figure S4 Altered FUT4 mediated PI3K/AKT/mTOR pathway in CRC cells (A) FUT4 or siFUT4 regulated the activity of PI3K/Akt/mTOR pathway by western blot. (B) The activity of PI3K/Akt/mTOR pathway was measured in SW480 cells treatment with Exo-SW620, FUT4 or siFUT4. [file 13046_2020_1562_MOESM4_ESM.tif]

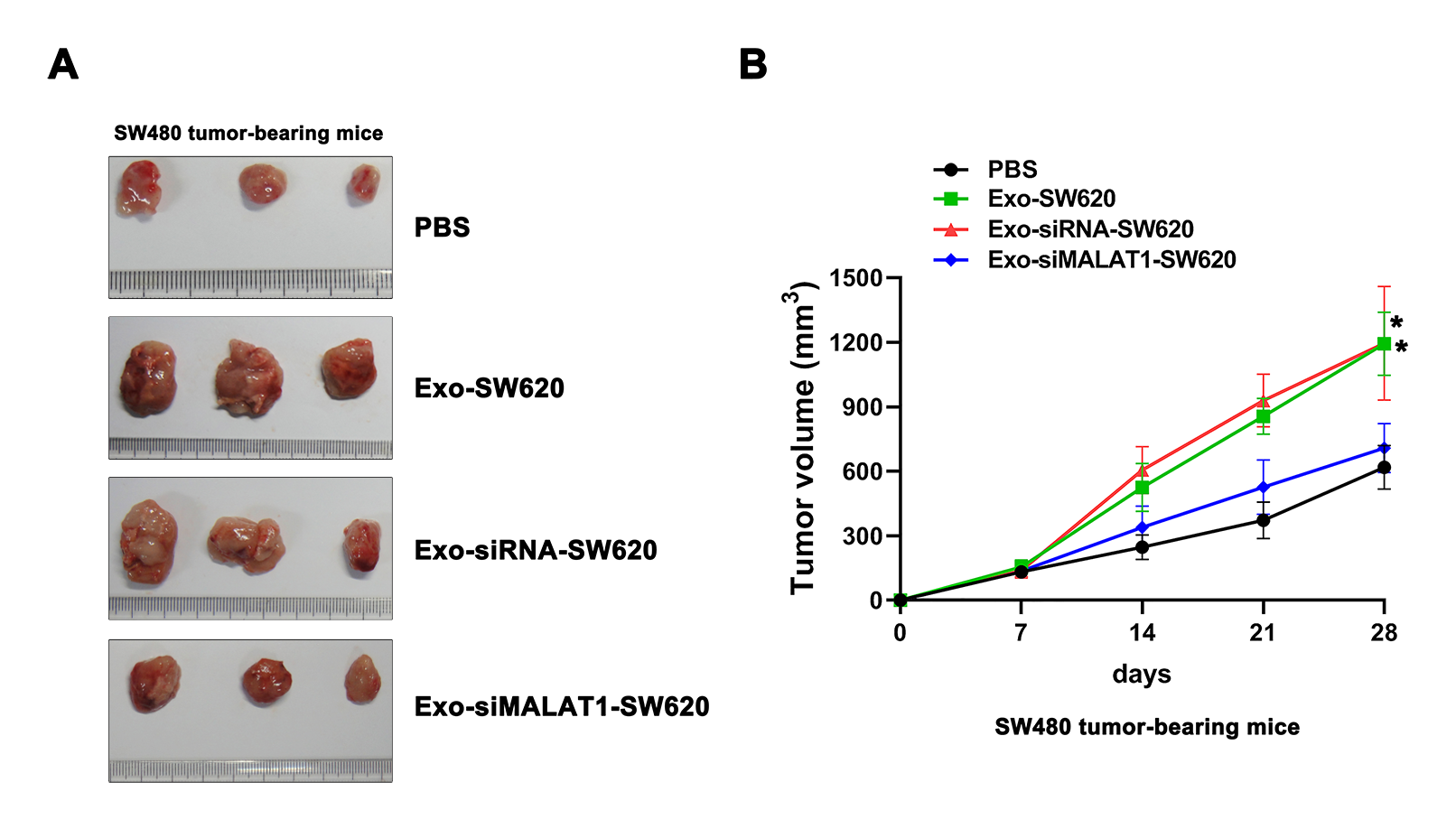

Supplement: Supplementary file 5 — Additional file 5 Figure S5 The effect of intratumorally injected exosomes on CRC tumor growth (A) The mice models were randomly assigned to four groups and inoculated subcutaneously with equal number of SW480 cells. Then from the seventh day on, PBS or 10 μg exosomes were intratumorally injected into tumor-bearing mice twice a week. 21 days after injection, mice were sacrificed and tumors were isolated and weighed. (B) The tumor size was measured every 7 days and tumor volume was calculated to assess the promotional effects of exosomes on CRC progression. Data were means ± SD of three independent assays (*P < 0.05). 9. [file 13046_2020_1562_MOESM5_ESM.tif]

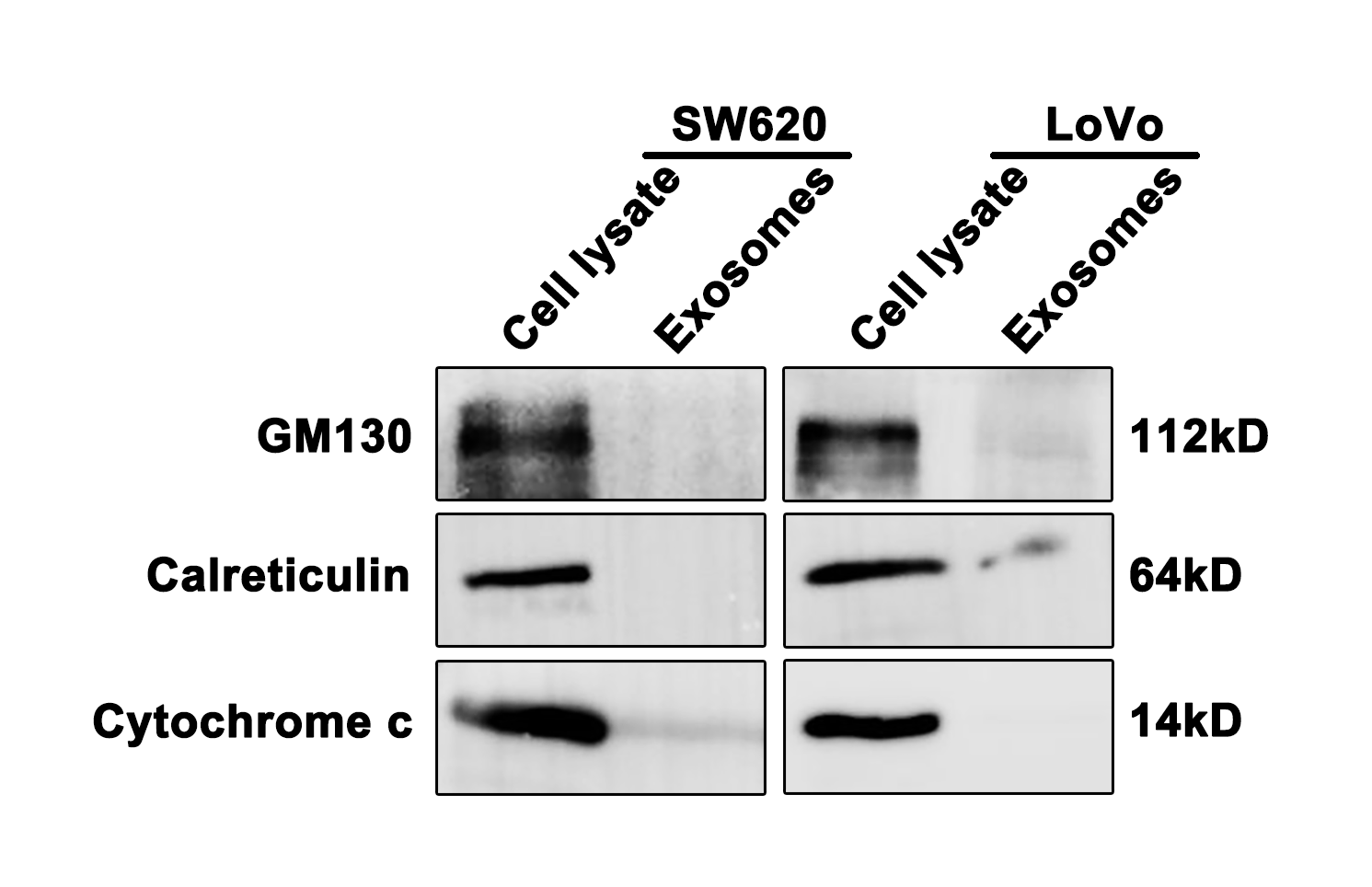

Supplement: Supplementary file 6 — Additional file 6 Figure S6 The protein levels of GM130, Calreticulin and Cytochrome c in exosomes from CRC cells and cell lysates were analyzed. Western blot was used to detect GM130 (Golgi marker), Calreticulin (endoplasmic reticulum marker) and Cytochrome c (mitochondria marker) expression in CRC exosomes and cell lysates with equivalent protein amount. [file 13046_2020_1562_MOESM6_ESM.tif]
